# Residual decontamination chemical agents negatively affect adhesion and proliferation of osteoblast-like cells on implant surface

**DOI:** 10.1186/s40729-020-00278-8

**Published:** 2020-12-17

**Authors:** Ísis de Fátima Balderrama, Matheus Völz Cardoso, Vitor Toledo Stuani, Rodrigo Cardoso Oliveira, Adriana Arruda Matos, Sebastião Luiz Aguiar Greghi, Adriana Campos Passanezi Sant’Ana

**Affiliations:** 1 Department of Diagnosis and Surgery, Araraquara School of Dentistry, Sao Paulo State University, Araraquara, Sao Paulo Brazil; 2 Department of Prosthodontics and Periodontics, Bauru School of Dentistry, University of Sao Paulo, Bauru, Sao Paulo Brazil; 3 Department of Biological Science, Bauru School of Dentistry, University of Sao Paulo, Bauru, Sao Paulo Brazil

**Keywords:** Dental implants, Decontamination, Osteoblast-like cells, Implant surface, Peri-implantitis

## Abstract

**Purpose:**

To investigate the influence of implant surface decontaminated and uncontaminated on osteoblast-like cell adhesion and proliferation

**Materials and methods:**

Commercially available implants of different brands and surface characteristics were selected: *Biomet 3i®* Nanotite (NT) and Osseotite (OT)*, Straumann®* SLActive (SLA), and *Neodent®* Acqua Drive (ACQ) and Neoporos Drive CM (CM)*.* Physical and chemical properties of the implants were investigated by scanning electron microscopy (SEM), energy dispersive X-ray spectroscopy (EDS), and wettability analysis (WETT). Implants were previously contaminated with *Aggregatibacter actinomycetemcomitans* strains; after that, samples were decontaminated by different chemical methods. Decontaminated (test group; *n* = 15/type of implant) and uncontaminated (control group; *n* = 5/type of implant) samples were analyzed according to the number of human osteoblastic osteosarcoma cells (Saos-2) adhered on the implant surface after 24 h and 72 h in SEM images.

**Results:**

ACQ was found to be highly hydrophilic, and NT was the most hydrophobic implant. Increased variation of Saos-2 cell adhesion and proliferation were observed on all test and control groups. Controversially, at the proliferation analysis in 72 h, CM implant was the only implant that showed no significant difference between test and group (*p* = 0.2833; Tukey’s multiple comparisons test). NT implants showed the greater value of cell proliferation when compared with all types of implant surface (*p* = 0.0002; Tukey’s multiple comparisons test).

**Conclusions:**

These findings suggest that decontaminated surfaces were able to impair the counting of osteoblast-like cell adhesion and proliferation.

## Introduction

Osseointegration is defined as a direct contact between vital bone and implant surface at optical microscopy level [1] and describes a rigid fixation of an alloplastic material in the bone, in an asymptomatic clinical situation and functional load [2]. The literature demonstrates five factors that are essential for osseointegration, such as implant design, implant material, surface properties, bone quality, and surgical technique [3]. Among surface properties, topography, chemistry, charge, and wettability are key determinants for osseointegration [4]. Changes on implant surface properties, such as an increase in roughness, were proposed to lead to a better clinical and histological result [5], influencing cell behavior [6] and biofilm formation [7].

Peri-implant microbiota has a similar composition to the one observed at the gingival sulcus of adjacent teeth [8–10]. Just like in natural teeth, the development of biofilm induces biological responses of hard and soft tissue around implants, resulting in pocket deepening, bleeding on probing, exudation, bone loss, and clinical mobility, impairing the success of implant therapy [8, 11].

The colonization, structure, and composition of the biofilm on implant surfaces is influenced by its surface roughness, chemical composition, hydrophobic properties, surface electrical charge, and energy [7, 12]. Modifications in micro- and nano-topography of dental implants were proposed to increase bone-to-implant contact, but biofilm accumulation in rougher surfaces is accelerated when implant threads are exposed to the oral cavity [12, 13], which make these areas more difficult to decontaminate [13]. On the other hand, moderate rough implants show higher rates of re-osseointegration when compared to machined ones [14].

Considering that decontamination of rough implant surfaces is a challenge in the treatment of peri-implant diseases, it is essential to provide adequate conditions to obtain re-osseointegration [15–17] by removing all the contaminant load, since bacterial remnants impair re-osseointegration and leads to peri-implantitis recurrence [18]. Decontamination and/or detoxification of implant surfaces can be performed by mechanical and chemical methods [17–20]. There is no consensus in literature on the best protocol to decontaminate implant surfaces [21]. Decontamination with different solutions was unable to completely decontaminate implant surfaces [22, 23] and could induce changes in the chemical and physical properties of implant surfaces [24], with partial re-osseointegration being reported in animals [22].

The aim of this study is to investigate the behavior of osteoblastic cells in different moderate rough implants and compare the results of different pristine and decontaminated surfaces.

## Material and methods

### Experimental design

**Fig. 1:**
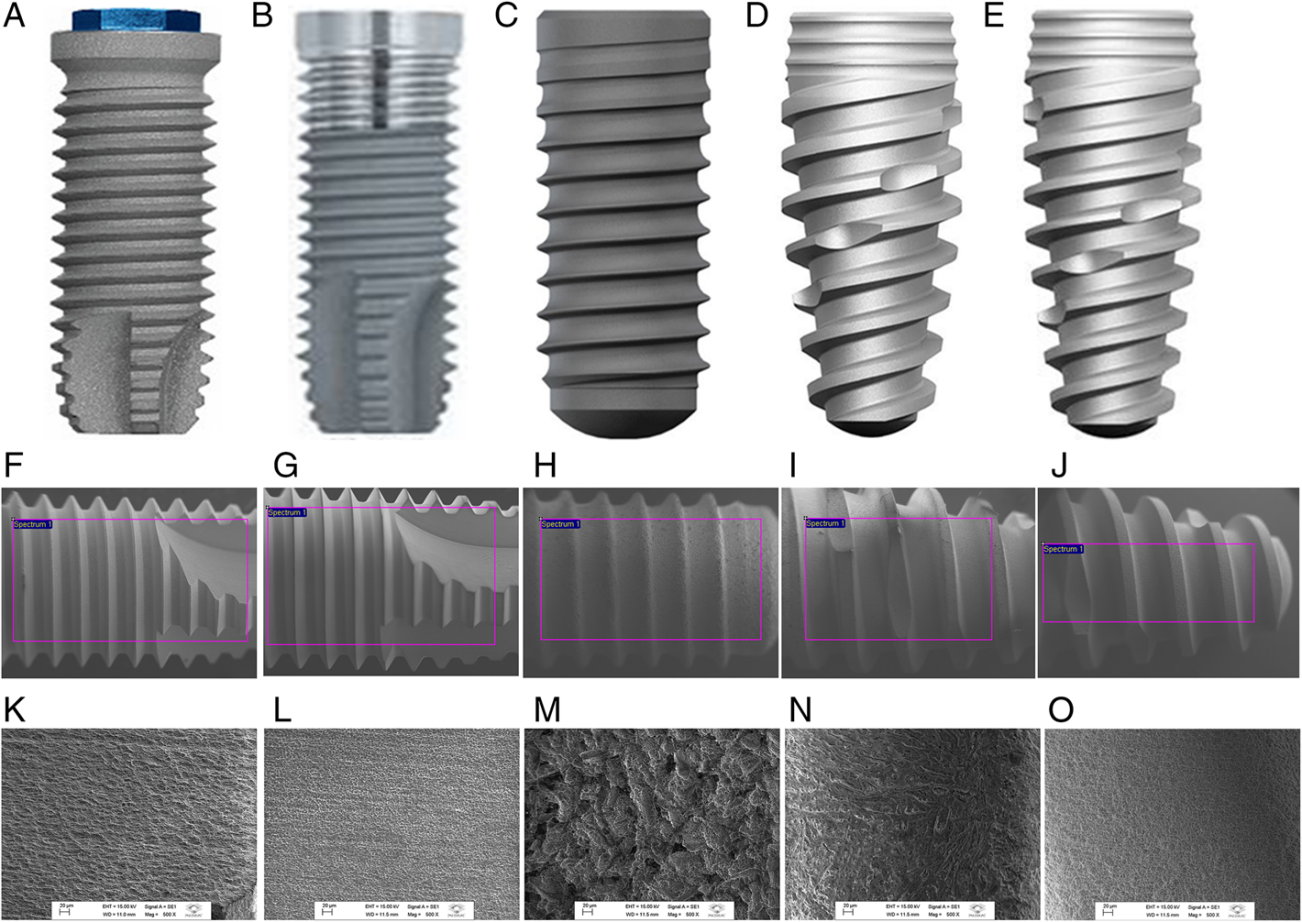
General features and macrogeometry of each model of dental implant evaluated (Macrogeometry and SEM images; × 500 magnification). **a**, **f**, **k** Nanonite, Biomet 3i®, USA. **b**, **g**, **l** Osseotite, Biomet 3i®, USA. **c**, **h**, **m** Bone Level, Sraumann®, Switzerland. **d**, **i**, **n** Drive CM Acqua, Neodent®, Brazil. **e**, **j**, **o** Drive CM Neoporos, Neodent®, Brazil

Five types of implants with different macro and microstructure were acquired in the market and are described in Table 1 and Fig. 1.

**Table 1:** Characteristics of dental implants evaluated according to manufacturer

Manufacturer	Implant type	Surface treatment	Macrogeometry	External dimensions (Ø × L)
1.Biomet 3i® (USA)	Nanotite	Dual-acid-etched and nanometer scale crystals of calcium phosphate	Parallel walled Tapered	4.0 × 10 mm
2.Biomet 3i® (USA)	Osseotite	Dual-acid-etched, sandblasted and processed with nitric, hydrochloric and sulfuric acids	Parallel walled Certain Tapered	4.0 × 10 mm
3. Straumann® (Switzerland)	Bone level	SLActive, chemically modified surface of sandblasted, large-grit, acid-etched	Hybrid tapered, platform switch	4.1 × 10 mm
4. Neodent® (Brazil)	Drive CM Acqua	Acqua, sandblasted, acid-etched and immersed in 0.9% sodium chloride	Conical central, Cone Morse connection	4 3 × 10 mm
5.Neodent® (Brazil)	Drive CM Neoporos	Neoporos, sandblasted with acid-etched	Conical central, Cone Morse connection	4.3 × 10 mm

### Surface chemical composition

The chemical composition of each implant surface (*n* = 1/type of implant) was investigated at cervical, central, and apical regions by energy dispersive X-ray detector (EDS, INCA x-act, Oxford) to analyze the percentage of chemical atomic element at the Anelasticity and Biomaterials Laboratory of Sao Paulo State University (Bauru, Sao Paulo, Brazil).

### Wettability (sessile-drop contact angle measurements)

**Fig. 2:**
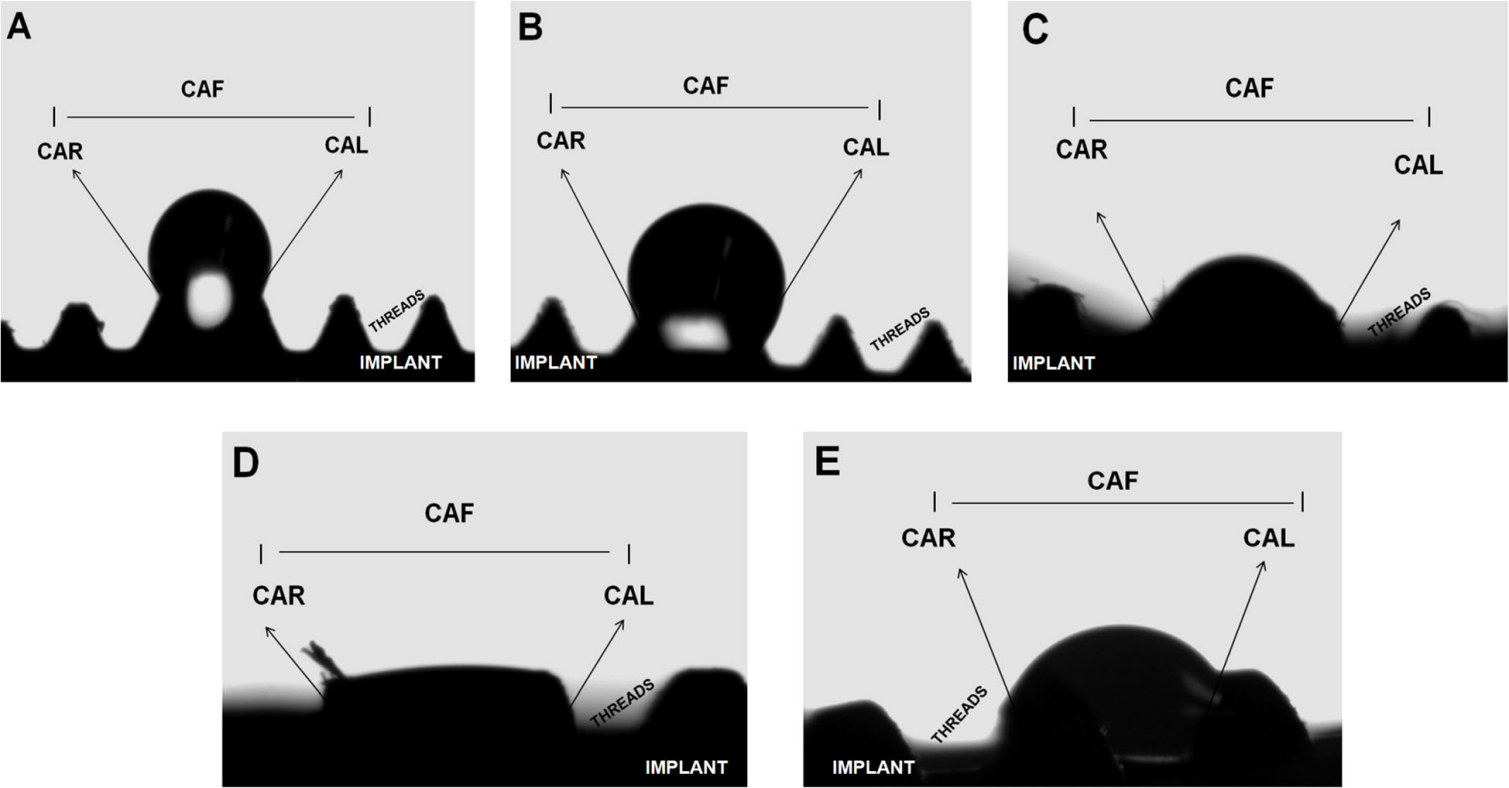
Wettabilty analysis. CAR, contact angle right side; CAL, contact angle left side; CAF, contact angle final; **a** Nanotite. **b** Osseotite. **c** SLActive. **d** Acqua. **e** Cone Morse Neoporos

Wettability properties were investigated on implant surface (*n* = 1/type of implant) at the Chemical-Physical Department of São Paulo State University (Araraquara, Sao Paulo, Brazil). Samples were stabilized in acrylic resin models and analyzed only in the mid-third of each implant. Wettability was analyzed at room temperature, with 75% air relative humidity, at the Contact Angle System (video-based Dataphysics, OCA, 15model). Average values of the right and left angles of each sample were determined. Standardized parameters were adjusted in a volume water of 1.000 μl, a medium probe, and a wettability time (waterfall) of 10 s. The right and left contact angle (CA) were defined by Young La Place equation (Fig. 2).

### Contamination of implants with strains of *Aa (*ATCC 29523)

The protocol of contamination was based on Freire et al. [25]. An amount of 20 μl of *A. actinomycetemcomitans* (Aa) was prepared, and after 2 days of incubation, the colony was collected and transferred in a tube with 5 mL of broth culture medium BHI (Brain Heart Infusion Agar, Acumedia, Neogen Corporation, MI, USA) at 37 °C for a period of 4 days. Implants were contaminated with a bacterial concentration of 1.5 × 10^8^ CFU in 2 mL of broth culture medium per well.

### Decontamination of implant surfaces

After the days of contamination process, the decontamination of implant surface was performed inside a laminar flow hood. The groups of different protocols for decontamination were prescribed for the sample (*n* = 6/type of implant):

1. Immersion in chlorhexidine 0.12% for 3 min;
2. Sequential immersion in EDTA 24% for 3 min and citric acid 10% for 3 min;
3. Antimicrobial photodynamic therapy (methylene blue 100 μg/ml and Diode LASER, Therapy XT [DMC, Sao Carlos, Sao Paulo, Brazil] for 3 min).

In the final stage, all implants were rinsed with saline solution for 1 min. The experimental group consists of the sum of all decontaminated samples, and the control group consists of implant surface uncontaminated (*n* = 1/type of implant).

### Osteoblast-like cell adhesion and proliferation

**Fig. 3:**
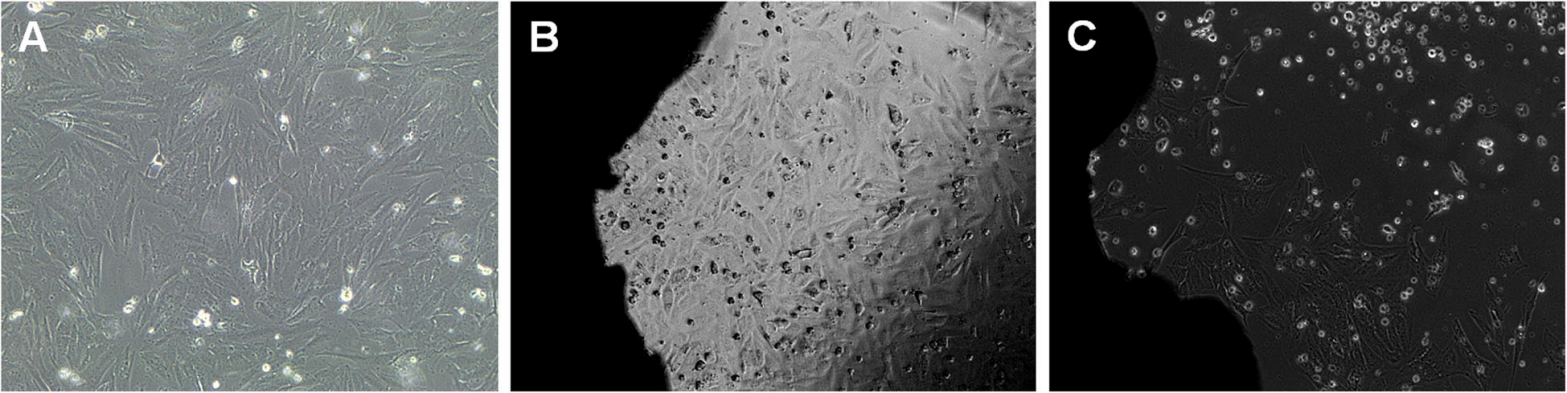
Human primary osteogenic sarcoma (Saos-2) cells (5 × 10^4^ cells). **a** Saos-2 cells before plating; **b** 24 h of Saos-2 cells on dental implant surface; **c** 72 h of Saos-2 cells on dental implant surface

Pre-existing human primary osteogenic sarcoma (Saos-2) cells (ATCC HTB85) were gently provided by the Department of Oral and Maxillofacial Surgery and Periodontics of Ribeirao Preto School of Dentistry—USP. Cells were cultivated in 15% McCoys’ culture medium containing 2 mM glutamine and 10% fetal bovine serum in humid atmosphere containing 5% CO_2_ at 37 °C. Culture medium was replaced every other day. Upon confluence, cells were detached by enzymatic methods (0.2% trypsin) and transferred to progressively larger tissue flasks (Sigma-Aldrich, Sao Paulo, Brazil). Saos-2 cells (5 × 10^4^ in 220 μL of culture medium) were plated on pristine implants (control; *n* = 5/type of implant) and on decontaminated implants (test; *n* = 15/type of implant). For this, the samples were positioned in 24-well cell culture plates containing culture medium 15% McCoy’s 5A + 2 mM glutamine + 10% fetal bovine serum (medium = 50 ml, 42.5 McCoys more 7.5 fetal bovine saline), and were maintained in an incubator for wet cell culture atmosphere containing 5% CO_2_ at 37 °C (Fig. 3). After 24 h and 72 h, the samples were fixed with Karnovsky solution (6% glutaraldehyde and 4% paraformaldehyde in 0.2 M cacodylate buffer) for 2 h. After two washes in 0.1 M cacodylate buffer for 15 min, the specimens were fixed with 2% osmium tetroxide in cacodylate buffer at 4 °C for 2 h and dehydrated with solutions of alcohol 30–70%. After gradually increasing concentration gradient, the specimens were embedded in 100% hexamethyldisilazane and dried at room temperature for 24 h. After that, the specimens were coated with gold (Q150R E Plus, UK) for 20 s with a density of 9.32 g/m^3^. Three photomicrographs corresponding to the cervical, central, and apical thirds of each implant investigated were acquired at × 500 magnification (AFORE, JEOL, JSM-6610, Japan). The adhesion of Saos-2 cells on implant valleys and flanks were analyzed in combination. Cell counting was performed using the ImageJ software (NIH, Bethesda, USA), according to the plugin Counting Cells (University of Chicago, USA). Briefly, SEM images were converted to 32 bits; the threshold tool was selected to subtract background and analyze particles, resulting in the total and percentage area covered by cells.

### Statistical analysis

Statistical analysis was performed at GraphPad Prism 7.0 for Mac, adopting a 5% significance level at all tests. Comparisons in wettability analysis were performed by Kruskal-Wallis post hoc Dunn. The number of cells adhered to implant surfaces after 24 h and 72 h in experimental and control groups were analyzed between and within groups by ANOVA post hoc Tukey’s multiple comparisons test.

## Results

### Surface chemical composition

**Fig. 4:**
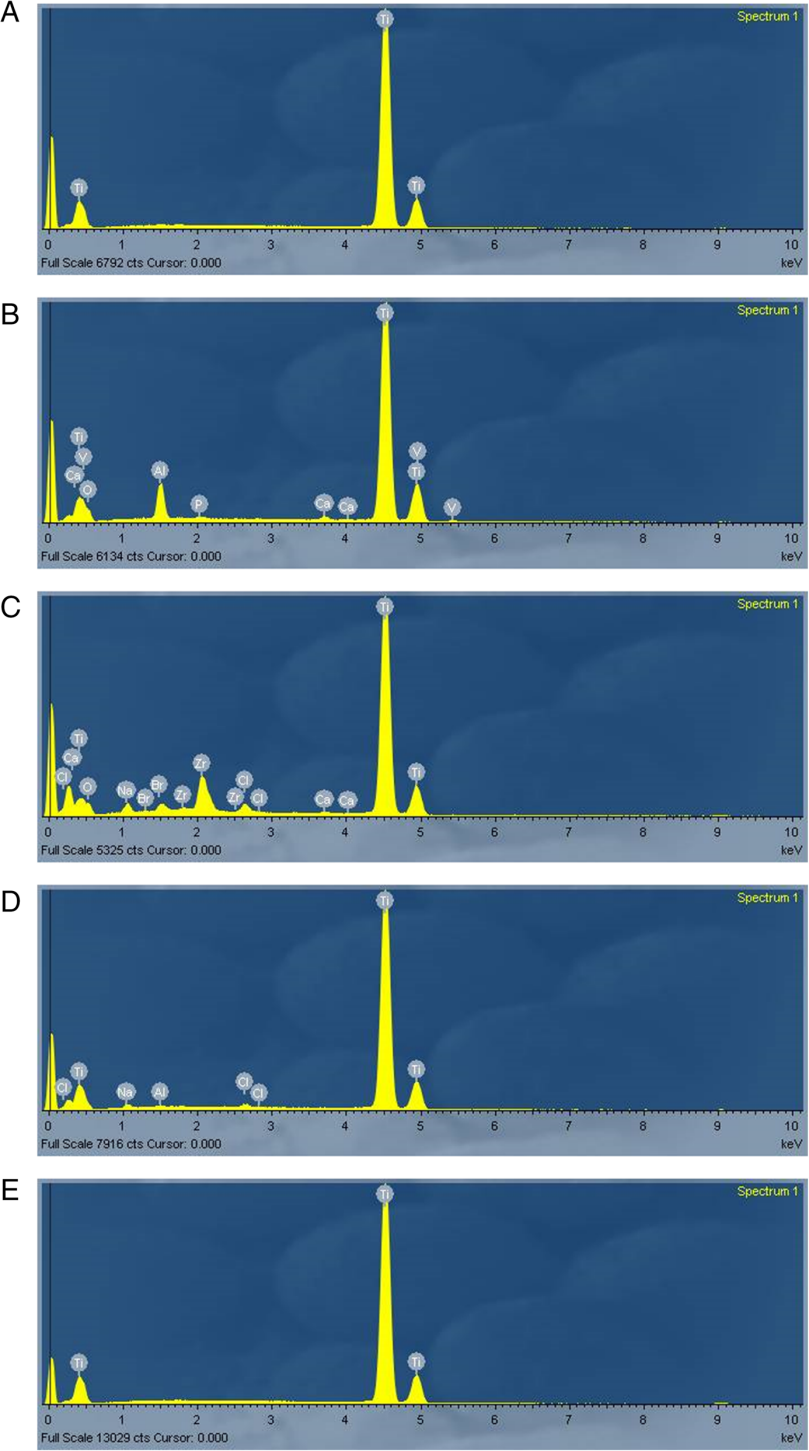
Spectrum of EDS for the area analyzed. Chemical composition of titanium with different surface treatment, note the high peaks of titanium element (**a**, **b**, **c**, **d**, and **e**). **a** Nanotite. **b** Osseotite. **c** SLActive. **d** Acqua. **e** Cone Morse

The chemical composition in atomic percentage (atomic%) of each implant is demonstrated in Fig. 4. NT and CM implants were composed of titanium only (100%); OT implant showed the presence of titanium (65.86%), oxygen (23.92%), aluminum (6.21%), vanadium (2.92%), calcium (0.69%), and phosphorus (0.40%); SLA implant showed the presence of titanium (66.38%), oxygen (22.73%), zirconium (5.12%), sodium (3.11%), bromine (0.77%), chlorum (1.60%), and calcium (0.30%); and ACQ implant showed the presence of titanium (97.34%), sodium (1.54%), chlorum (0.78%), and aluminum (0.33%).

### Wettability analysis

The sessile-drop contact angle analysis showed that ACQ exhibited the most hydrophilic behavior, while NT and OT exhibited more hydrophobic responses (Table 2). Significant differences (*p* < 0.0001; Kruskal-Wallis) were found between NT (hydrophobic) and ACQ (hydrophilic).

**Table 2:** Wettability results

	CAR	CAL	CAF
NT	126.6°	126.3°	131.1°*
OT	115.4°	117.2°	110.9°
SLA	55.9°	60.0°	40.6°
ACQ	< 5°	< 5°	< 5°*
CM	76.3°	73.6°	79.4°

*Significant differences (*p* < 0.05)

Kruskal-Wallis *p*< 0.0001; significant differences (*p*<0.05) between groups NT and ACQ (mean values)

### Adhesion and proliferation of Saos-2 cells in uncontaminated and decontaminated sites

**Fig. 5:**
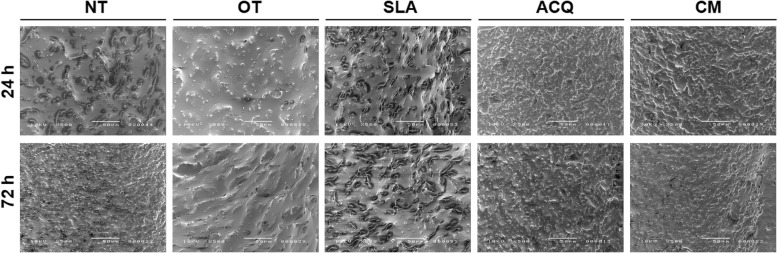
SEM photomicrographs of Saos-2 cell adhesion and proliferation. SEM images of Saos-2 cells on NT, OT, SLA, ACQ, and CM dental implant surfaces after 24 and 72 h, adhesion and proliferation respectively (× 500 magnification)

All decontaminated implants showed less cells adhered at surfaces after 24 h and 72 h when compared to the respective uncontaminated sites (control group) (Table 3). The results of cell adhesion on implant surface at 24 h (Fig. 5) after decontamination protocols (test group) showed lower values when compared to the control group; however, only CM implants showed statistic significant difference between test and control group (*p* = 0.0498; Tukey’s multiple comparisons test) (Table 3).

**Table 3:** Cell adhesion (24 h) and proliferation (72 h) on implant surfaces (mean ± SD)

	NT			OT			SLA			ACQ			CM		
	Control	Test	***p***	Control	Test	***p***	Control	Test	***p***	Control	Test	***p***	Control	Test	***p***
**24 h**	4535.3 ± 2919.5a,c	1329.8 ± 205.7a,b	*> 0.9999*	5140.0 ± 929.8c	2468.1 ± 658.7a,c,b	*0.3205*	3538.3 ± 2251.9a,c,b	864.6 ± 445.0a,b	*0.3197*	4109.6 ± 843.1a,c,b	1688.6 ± 326.2a,b	*0.4444*	4519.6 ± 559.2a,c	746.2 ± 863.2b	*0.0498*
**72 h**	14,368.7 ± 3481.8a	2160.7 ± 2912.0b	*< 0.0001*	7694.3 ± 655.3 c	1446.8 ± 725.9 b	*0.0026*	7165.6 ± 122.0 c	642.0 ± 190.6 b	*0.0016*	8831.3 ± 402.5 c	2669.7 ± 562.5 b	*0.003*	6558.6 ± 1285.3 c	3267.3 ± 689.7 b	*0.2833*
***p***	*0.02*	*0.6479*		*0.0177*	*0.1455*		*0.0495*	*0.4703*		*0.0009*	*0.0592*		*0.0654*	*0.0168*	

Tukey’s multiple comparisons test; post hoc Tukey; different letters in same line represent significant differences between groups

*C* control (uncontaminated), *T* test (decontaminated)

*Significant differences (*p* < 0.05); Tukey’s multiple comparisons test

The results of cell proliferation on implant surface at 72 h (Fig. 5) after decontamination protocols (test group) also showed lower values when compared to pristine implants (control group). No differences were found between intergroup analysis in the test group (*p* > 0.05; Tukey’s multiple comparisons test). NT implants showed the higher value at cell proliferation for the control group, showing statistic significant difference when compared to all other types (*p* = 0.0002; Tukey’s multiple comparisons test) (Table 3). Therefore, when comparing the control with the test group, only CM showed no significant difference (*p* = 0.2833).

## Discussion

In the past few years, the prevalence of mucositis and peri-implantitis has increased, varying from 19 to 65% and from 1 to 47%, respectively [26]. Plaque accumulation at implant surfaces triggers an inflammatory response that leads to the development of mucositis or peri-implantitis [27]. In this study, we have investigated surface properties of different implants acquired in the market, including chemical composition and wettability analysis. Additionally, we have also investigated the effects of chemical surface decontamination on the adhesion and proliferation of osteoblast-like cells. Implant surface properties are influenced not only by microtopography or nanotopography, but also by macrotopography. Most in vitro studies investigating the effects of decontamination methods of implant surfaces are performed in titanium disks [7, 12, 19, 21, 24, 28–33] which do not reproduce the macro- and microtopography characteristics of implants. For these reasons, in this study, we aimed to investigate the impact of chemical decontamination at cell adhesion and proliferation in five different dental implants of different brands that previously received unique surface treatments and are considered the most commercially available in the world.

Rosa et al. [34] have also investigated implants from different batches and companies, totalizing 3 implants per group, and found differences from batches of the same company in two types of Brazilian implants, while the remaining samples were more uniform. Considering that, we have used 6 samples of each implant, minimizing the risk of bias.

Microbial colonization around implants follows a similar course than natural teeth, with a shift in microbial composition as disease develops [35]. A study [36] showed that the prevalence of periodontal pathogens was similar among individuals with periodontitis and peri-implantitis, independent from health condition. While the prevalence and levels of *P. gingivalis* and *F. nucleatum* were positively associated with periodontitis, but not with peri-implantitis, *A. actinomycetemcomitans* was positively associated with both periodontitis and peri-implantitis. Different studies showed that *A. actinomycetemcomitans* is consistently found in peri-implantitis [10, 36–38], being able to infect the abutment-implant interface [9, 10]. In this study, a strain of *A. actinomycetemcomitans* was used to contaminate implant surfaces, considering that this species is able to induce biofilm formation in smooth and rough surfaces in vitro and to trigger an inflammatory response characterized by spontaneous bleeding, ulceration, soft tissue necrosis, hyperplasia, and implant failure in vivo [25].

Bacterial adhesion to Ti surfaces is roughness-dependent [7, 12], although not significantly influencing plaque composition [7]. Increased roughness provides a larger surface and additional niches for bacterial adhesion, reduces shearing forces, and, as a consequence, reduces the desorption of bacteria during early phases of adhesion [7, 39].

However, since in our study all implants were considered minimally to moderately rough, differences in the percentage of contaminated area may be attributed to surface properties other than roughness, such as surface energy, chemical composition, and wettability. All implants were composed of titanium (Ti), but OT, SLA, and ACQ showed the presence of chemical elements other than Ti, including aluminum, vanadium, sodium, chlorine, and oxygen. Chemical composition of the surface results in different reactions from the surrounding media [40] and is different from the bulk material due to preparation methods and impurities [41].

It has been shown that the chemical composition and surface topography in the macro and micro scales have strong effects on cell behavior [6]. Modifications in the nanoscale level, which can be created by deposition of a material at implant surface or by etching away part of a surface, as observed in some implants investigated in this study, may also influence cell and bacterial behavior [42]. Although the nanotopographic surface is chemically similar to the bulk, the surface itself may induce differences in surface chemistry or energy, such as an increase in hydrophobicity [42]. The strength of cellular adhesion to a nanorough substrate can be predicted by a mathematical model: for small surface energy, increases in surface roughness impairs cell adhesion; in moderate or intermediate surface energy, increases in surface roughness has a minor effect on cell adhesion; and in large surface energy, optimal roughness maximizes cell adhesion [43]. Considering that, surface topography and physico-chemical properties of materials may promote cell adhesion and influence bacterial adhesion [42].

These characteristics may be modified by mechanical and/or chemical treatments performed to detoxify contaminated implant surfaces. Therefore, it is important to determine if moderately rough implants decontaminated by mechanical and chemical methods allows the adhesion of osteoblastic cells, which is essential to re-osseointegration. Our findings showed that, despite decontamination procedures, all implants may have presented a remaining contaminated area that may have affected cell adhesion and proliferation, since pristine implants showed higher number of cells attached to the surface after 24 h and 72 h. Since no differences in the number of attached cells to hydrophobic or hydrophilic surfaces were found, it can be assumed that residual contamination impaired cell adhesion and spreading. Bürgers et al. [29] have also shown that decontamination of titanium discs previously contaminated by monocultures of *S. epidermidis*, *S. sanguinis,* and *C. albicans* were only partially effective, except for the use of sodium hypochlorite, which is toxic to patients.

Hydrophilic surfaces tend to enhance the early stages of cell adhesion, proliferation, differentiation, and bone mineralization [44, 45], and promote earlier osseointegration as determined by greater bone-to-implant contact at initial stages of osseointegration [46]. Surface energy of an implant, indirectly measured by the liquid-solid contact angle (CA), is therefore related to wettability [47].

In this study, three surfaces were considered as hydrophilic (SLA, ACQ, and CM), and the remaining two (NT and OT) were found to be hydrophobic. ACQ was considered as superhydrophilic, since the angle contact is close to 0^o^. ACQ showed the less percentage of contaminated area, with significant differences from NT. Rodriguez Y Baena et al. [48] showed significantly less contaminated area in OT and even lesser on NT than on machined implants, especially for *A. actinomycetemcomitans*, *S. mutans*, and *S. sanguis* than for *P. gingivalis* and *S. salivarius* strains. Similar results were described by Lubin et al. [30], who showed that OT discs were easier to decontaminate than NT and that tetracycline and citric acid were the most effective solutions for the disinfection of *P. gingivalis* from OT discs. These findings suggest that different solutions may have different outcomes depending on surface characteristics.

## Conclusions

Only NT and CM implants showed chemical composition by titanium pure. ACQ exhibited hydrophilic behavior while NT and OT hydrophobic properties. The decontaminated implants showed lesser cells adhered during 24 and 72 h when compared to uncontaminated implants, corresponding respectively to adhesion and proliferation analysis. CM implants showed more cells attached after 24 h for experimental group. NT implants demonstrated more cell proliferation after 72 h for control group. Considering that, further preclinical and clinical studies are necessary to determine how different decontamination protocols can affect cell migration and proliferation on implants with different surface treatment, chemistry, surface energy, and wettability.

## Data Availability

The authors from this work are available to support data.

## Abbreviations

NT: Nanotite
OT: Osseotite
SLA: SLActive
ACQ: Acqua
CM: Neoporos drive Cone Morse
SEM: Scanning electron microscopy
EDS: Energy dispersive X-ray spectrometry
WETT: Wettability analysis
Saos-2: Pre-existing human primary osteogenic sarcoma cells
*Aa*: *A*ggregatibacter actinomycetemcomitans
EDTA: Ethylenediaminetetraacetic acid
CA: Contact angle
